# Platelet Parameters and (1, 3)-β-D-Glucan as a Diagnostic and Prognostic Marker of Invasive Fungal Disease in Preterm Infants

**DOI:** 10.1371/journal.pone.0123907

**Published:** 2015-04-13

**Authors:** Dongying Zhao, Gang Qiu, Zhongcheng Luo, Yongjun Zhang

**Affiliations:** 1 XinHua Hospital, Shanghai Jiao-Tong University School of Medicine, Shanghai, China; 2 MOE and Shanghai Key Laboratory of Children’s Environmental Health, Shanghai Jiao-Tong University School of Medicine, Shanghai, China; 3 Shanghai Children's Hospital, Shanghai Jiao-Tong University School of Medicine, Shanghai, China; Centre Hospitalier Universitaire Vaudois, FRANCE

## Abstract

The diagnosis of neonatal invasive fungal disease (IFD) is difficult and often delayed. The platelet parameters and (1, 3)-β-D-Glucan (BG) may be useful for diagnosing IFD, but their diagnostic performance are not well characterized in neonates. We studied 63 preterm infants with IFD, 160 preterm infants without sepsis (preterm control), and 41 preterm infants with bacterial sepsis. Platelet parameters at the first day of onset of IFD and at the fourteenth day after antifungal treatment were evaluated. Serum BG was measured. Platelet count (PC), plateletcrit (PCT), and platelet distribution width (PDW) values were significantly lower, and mean platelet volume (MPV) values significantly higher in the IFD versus preterm control infants. PC and PCT values were much lower in infants with IFD versus bacterial sepsis, and there were significant differences in BG value between the two groups. After 14 days of antifungal treatment, significant elevations in PC, PCT, PDW and reductions in MPV levels in IFD group were observed. Receiver operating characteristic (ROC) curves showed that PC and PCT were strong predictors of IFD. The PC and PCT cut-offs for predicting IFD were 119.5 (sensitivity 78%, specificity 95%) and 0.21 (sensitivity 83%, specificity 85%), respectively. There were significant differences in PC and PCT levels between deceased and survived patients. The PC and PCT cut-offs for predicting deceased IFD were 39 (sensitivity 62%, specificity 86%) and 0.04 (sensitivity 50%, specificity 95%), respectively. The sensitivity in diagnosing IFD by a BG cutoff of ≥10pg/ml was 68.3% and specificity was 75.6%. PC and PCT levels in the BG ≥400 pg/ml group were significantly lower compared to the BG<400 pg/ml group. Platelet parameters and BG could be useful biomarkers for the diagnosis and prognosis of neonatal IFD.

## Introduction

Invasive fungal disease (IFD) is an important cause of late-onset sepsis in the neonatal intensive care unit (NICU), and is associated with severe co-morbidity and high rates of neurodevelopmental impairment [1–3]. The diagnosis of neonatal IFD is difficult and often delayed because of its polymorphic clinical presentations. Currently, a positive blood culture for candida species remains the gold standard in diagnosing IFD. However, this diagnostic test may take several days. In addition, blood culture has been shown with poor sensitivity for the diagnosis of IFD [4, 5]. Dependence on blood culture results can therefore result in under-diagnosis of fungal infection and delay in the diagnosis and initiation of antifungal therapy.

Identification of diagnostic and prognostic factors that are available early during the disease course would improve our ability to individualize antifungal strategies. Non-culture-based diagnostic techniques for detecting IFD are therefore increasingly used to facilitate timely diagnosis. One of the developed biomarkers is (1, 3)-β-D-Glucan (BG), a major cell wall component of almost all fungi, is present on the surface of all Candida spp [6, 7]. BG detection has been widely used as a diagnostic tool for invasive fungal diseases in adults [6, 8]. The relationship between BG levels and treatment responses in premature infants with IFD has not been well characterized. We therefore sought to evaluate the diagnostic performance and prognostic usefulness of BG assay in neonatal candidiasis. Additionally, several lines of evidence support a role for platelets in inflammation [9]. Platelet-mediated inflammation has been demonstrated during various acute and chronic infections, and it has been suggested that platelets contribute to antimicrobial defense [10]. Platelets contribute to inflammation and promote thrombosis, characteristically seen in aspergillosis, and might be involved both in antifungal defense and in the histopathological process [11]. Thus, in addition to clinical symptoms, it has been indicated that platelet count (PC) and indices including plateletcrit (PCT), platelet distribution width (PDW), and mean platelet volume (MPV) might be useful together with other acute phase reactants to define inflammation activation during IFD.

The primary objective of the present study was to evaluate the sensitivity and specificity of serum BG, PC and platelet indices in the diagnosis and prognosis of IFD in premature infants in NICU and to analyze their changes during antifungal therapy.

## Materials and Methods

### Ethics and Clinical populations

This is a case control study of preterm infants admitted to 2 NICUs in Shanghai, including Xinhua Hospital and Shanghai Children`s Hospital, which are level-3 tertiary NICUs in China. The study was approved by the Research Ethical Committees of both hospitals. All parents or legal guardians of recruited infants signed an agreement after being disclosed to necessary information on this study, and had given written informed consent.

From January 2008 to June 2014, we identified 63 preterm infants with IFD, 160 age and gender matched preterm infants without sepsis (preterm control), and 41 preterm infants with bacterial sepsis (common bacterial infection control). Diagnosis of IFD was based on international consensus guidelines [12, 13]. In our study, all IFD infants were alive at 72 hours after birth, and had a positive blood culture for candida. Overall IFD mortality was defined as death prior to discharge or death within 7 days after discharge from NICU if no other pathogen was isolated from blood or from other clinical specimens and if clinicians documented that the infection or sepsis caused the death [14]. The preterm control included preterm infants with duration of hospital stay ≥21days. The definition for neonatal bacterial sepsis was according to the 2010, European Medicines Agency (EMA) meeting, which required the presence of at least two clinical symptoms and at least two laboratory signs in the presence of or as a result of suspected or proven infection (positive culture, microscopy or polymerase chain reaction) [15]. In our study, all the preterm infants with bacterial sepsis had an infection occurred at more than 1 week post partum and had a positive blood culture for bacteria. When these preterm infants were discharged from hospital after recovery, they had 30 days of clinical follow-up. Infants with congenital surgical anomalies or congenital infection and those lost to follow-up were excluded from the analysis. And all preterm infants had a normal level of platelet count at their first day of life.

During hospitalization, for IFD infants and preterm infants with bacterial sepsis, peripheral blood test and BG test would be performed once a week since the onset of the disease. Additionally, in the IFD infants, after the antifungal treatment, BG test would last until it became negative, and blood test would last until the patient was discharged. For preterm control, a routine peripheral blood test would be conducted at about once a week after birth, and a BG test would be done at about three weeks of postnatal life.

Full medical history, including demographical information, complete blood count (white blood cell count [WBC], hemoglobin [HB] and platelet parameters [including PC, PCT, PDW, MPV]) at the first day of onset of IFD and at the 14th day after antifungal treatment, BG measurements at the first day of onset of IFD or bacterial sepsis, the duration from BG positive to negative in IFD group, the underlying disease, duration of hospital stay before the onset of infection and microorganisms identified in blood specimen, was recorded. For comparisons, complete blood counts and BG value of preterm control at about postnatal age of 21 days or at the onset of bacterial sepsis were documented.

Some diseases could also affect platelet parameters such as asphyxia, necrotizing enterocolitis (NEC), respiratory distress syndrome (RDS), invasive mechanical ventilation, parenteral nutrition and the presence of central venous catheters (CVC) [16–19]. To prevent potential bias, we would balance these conditions among the 3 groups. Besides, we had collected information on some important underlying diseases including liver injury, heart diseases such as ventricular septal defect (VSD), patent ductus arteriosus (PDA), atrial septal defect (ASD) and fluconazole prophylaxis. The duration of parenteral nutrition was calculated as the days from the nutritional treatment to the onset /specimen collection days of IFD, bacterial sepsis and preterm control.

### BG Assay

Serum BG levels were measured by a GKT-5M Set Kinetic Fungus Detection Kit (Gold Mountainriver Techn Development Co.Ltd, Beijing, China). According to the manufacturer’s instructions, BG levels were automatically calculated based on a calibration curve with standard solutions ranging from 10 to 1000 pg/ml. BG values <10 pg/ml were interpreted as negative, >20 pg/ml as positive, and 10–20 pg/ml as uncertain.

### Fungal prophylaxis and Treatment

Fluconazole prophylaxis was used for all preterm infants with BW <1000 g and some preterm infants with BW <2000 g. Fluconazole was administered 3 mg/kg, every 48 hours, starting from day 3 of postnatal life. Infants were assigned to intervention for 14 days or until intravenous access was discontinued. When diagnosing an episode, removal of central vascular catheters was the standard policy for the management of central intravascular lines. All IFD episodes were treated with intravenous voriconazole at 6 (start) to 4 (steady state) mg/kg every 12 hours; the treatment was continued for at least two weeks after two negative cultures 48 hours apart.

### Statistical Analysis

All statistical analyses were performed using SPSS software, version 16.0 (SPSS Inc., Chicago, IL, USA). Comparisons of continuous data between groups used Student’s t-test or Mann–Whitney U test. For variables with normal data distribution, comparisons were made with the Student t-test or ANOVA. For variables with skewed data distribution, groups were compared with Kruskal-Wallis test or Mann-Whitney U test. Chi square test or Fisher exact test was used for comparisons of categorical variables. *P* values <0.05 were considered statistically significant.

## Results

### Characteristics of Preterm Patients in the Study Population

Among the 63 IFD infants, 7 (11.1%) were extremely low birth weight (ELBW<1000 g), 36 (57.2%) very low-birth-weight (VLBW, 1000–1499 g), and 20 (31.7%) low-birth-weight (LBW, 1500–2499 g). 22 (34.9%) were born at gestational age between 26 and 29 weeks, 35 (55.6%) between 30 and 33 weeks, and 6 (9.5%) between 34 and 36 weeks.

Mean age at the onset of IFD was 21.5±6.8 days. Asphyxia (33.3%) was the most common co-morbidity of IFD, followed by heart disease including VSD, PDA and ASD (27%). 21 (33.3%) patients required respiratory assistance with invasive mechanical ventilation. At the time of diagnosis of IFD, 44 (69.8%) patients had indwelling peripherally inserted central venous catheter. The mean duration of parenteral nutrition was 18.9±6.5 days in IFD.

Both IFD and bacterial sepsis patients had a significant higher overall mortality than preterm controls (*p*<0.001). Besides, analyses of other factors that could potentially affect platelet count-including asphyxia, NEC, RDS, invasive mechanical ventilation, parenteral nutrition and the presence of CVC-did not find any significant differences among the three groups (Table 1).

**Table 1:** Demographics, clinical characteristics and hematological findings in the study population.

Variable		IFD (n = 63)	Preterm control (n = 160)	Bacterial sepsis (n = 41)	*P* value
Demographics					
	Male	34 (54.0)	83 (51.9)	24(58.5)	0.74
	Gestational age (weeks)	30.7(2.3)	31.1(1.6)	31.0(2.2)	0.22
	Birth weight (g)	1485.1(444.5)	1498.9(258.6)	1613.7(389.3)	0.20
	Age at onset/collected day^a^	21.5(6.8)	20.9(1.4)	17.3(9.5)^c^ ^,^ ^d^	<0.001
	Mode of delivery (caesarean)	40 (63.5)	104 (65.0)	24(58.5)	0.75
Underlying diseases					
	Asphyxia	21(33.3)	43(26.9)	6(14.6)	0.11
	Bacterial infection	11(17.5)	44(27.5)	-	0.12
	Necrotizing Enterocolitis	6(9.5)	8(5)	6(14.6)	0.08
	Respiratory Distress Syndrome	11(17.5)	32(20.0)	7(17.1)	0.86
	Heart disease	17(27.0)	61(38.1)	19(46.3)	0.11
	Liver injure	10(15.9)	14(8.8)	7(17.1)	0.17
Antecedent neonatal events					
	Invasive Mechanical Ventilation	21(33.3)	67(41.9)	19(46.3)	0.36
	Duration of parenteral nutrition(days)	18.9(6.5)	17.1(3.8)	15.4(9.1)^c^ ^,^ ^d^	<0.001
	Fluconazole prophylaxis	29(46.0)	97(60.6)	23(56.1)	0.14
	Presence of CVC	44(69.8)	110(68.8)	28(68.3)	0.98
Overall mortality		13(20.6)	9(5.6)	8(19.5)	0.001
Hematological findings					
	HB (g/L)	120.1(27.3)	126.9(24.5)	128.4(34.2)	0.18
	WBC (×10^9^/L)	11.3(6.6)	10.9(3.5)	16.1(10.1)^c^ ^,^ ^d^	0.001
	PC (×10^9^/L)	95.8(81.6)	264.4(106.0)^b^	184.8(120.0)^c^ ^,^ ^d^	<0.001
	PCT (%)	0.13(0.14)	0.32(0.12)^b^	0.35(0.20)^c^	<0.001
	PDW (fl)	13.3(4.0)	15.7(3.6)^b^	13.7(3.9)	<0.001
	MPV (fl)	12.8(1.1)	12.1(1.0)^b^	12.7(1.9)^d^	<0.001

Data are presented as the number of subjects in each group, with percentages given in parentheses or mean, with SD given in parentheses.

^a^ Date were collected at the onset of IFD and bacterial sepsis, or about postnatal age of 21 days for preterm control infants.

^b^Comparison between IFD vs preterm control *p*<0.05

^c^Comparison between IFD vs bacterial sepsis *p*<0.05

^d^Comparison between bacterial sepsis vs preterm control *p*<0.05

### Isolates

Of the 63 IFD infants, isolated fungal species were as follows: C. albicans (17 cases), C. parapsilosis (21 cases), C. guilliermondii (18 cases), C. famata (6 cases) and C. krusei (1 case). Among the deceased IFD infants, there were 5 cases of C. albicans, 4 cases of C. parapsilosis, 2 cases of C. guilliermondii and 2 cases of C. famata.

In the bacterial sepsis group, gram positive and gram negative bacteria accounted for 17(41.5%) and 24 (58.5%), respectively. The most common gram positive organisms were Coagulase Negative Staphylococcus Aureus (CONS) 10(24.4%), while the most common gram negative organisms were E.coli 9 (22.0%) and Klebsiella pneumonia 8 (19.5%).

### Platelet Parameters in IFD, Preterm Control and Bacterial Sepsis

We compared hematological data among the 3 groups (Table 1), and observed differences statistically significant for WBC, PC, PCT, PDW and MPV values. PC, PCT and PDW values among infants at the onset of IFD were significantly lower and MPV value significantly higher than among preterm control infants. Furthermore, WBC, PC and PCT values in infants at the onset of IFD were significantly lower than in infants at the onset of bacterial sepsis.

**Fig 1:**
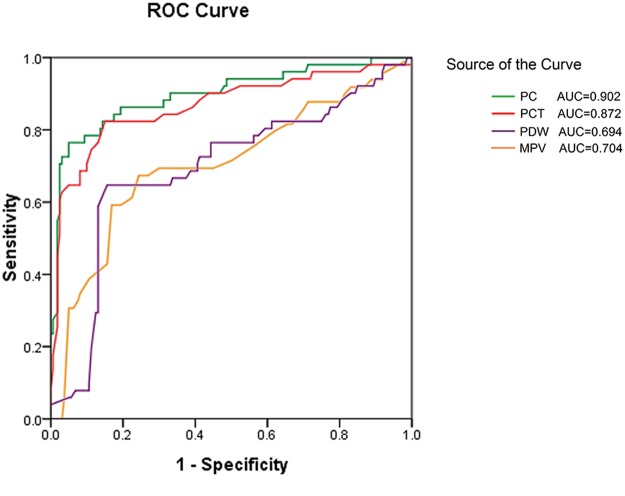
Receiver operating characteristic curves for the diagnosis of IFD by four platelet parameters. For each indicator, sensitivity (true positive rate) is plotted against 1-specificity (false positive rate). Accuracy is measured by the area under the ROC curve. Platelet count (PC) and plateletcrit (PCT) showed better accuracy than platelet distribution width (PDW) and hemoglobin (MPV).

Among patients with hemostatic biomarkers, we conducted Receiver Operating Characteristic (ROC) curve analysis to predict IFD. Fig 1 showed the ROC curves for the four hemostatic parameters. The AUCs in predicting IFD for PC (0.902) and PCT (0.872) were higher than those for PDW (0.694) and MPV (0.704). Table 2 showed that the best cut-offs for predicting IFD were 119.5 for PC (sensitivity 78%, specificity 95%) and 0.21 for PCT (sensitivity 83%, specificity 85%), respectively.

**Table 2:** ROC analysis for the prediction of IFD by platelet parameters using the cutoffs with the maximal sum of sensitivity and specificity.

	Cutoff value	AUC	95% Confidence interval	Sensitivity	Specificity	*P* value
PC (×10^9^/L)	119.5	0.902	0.851–0.953	0.78	0.95	<0.001
PCT(%)	0.21	0.872	0.807–0.937	0.83	0.85	<0.001
PDW(fl)	12.0	0.694	0.604–0.783	0.65	0.84	<0.001
MPV(fl)	12.8	0.704	0.613–0.795	0.67	0.76	<0.001

AUC = area under the curve

### Serum BG levels in Invasive Fungal Disease

In IFD infants, the median BG value was 208.2 pg/ml; BG level <416 pg/ml was correlated with treatment success in a previous study [6]. So, we grouped IFD patients by serum BG cutoff at 200 pg/ml or 400 pg/ml. There were 21 infants with serum BG >400pg/ml, and 31 infants >200 pg/ml in the IFD group, while there were only 2 neonates with serum BG >400pg/ml, and 4 neonates >200 pg/ml in the bacterial sepsis group. Table 3 showed there were significant differences in BG value between the IFD and bacterial sepsis at a cutoff 200mg/dl or 400mg/dl.

**Table 3:** IFD and bacterial sepsis by BG value.

	BG value (pg/ml)		*P* value	BG value (pg/ml)		*P* value
	<200	≥200		<400	≥400	
IFD(n)	32	31	<0.001	42	21	<0.001
Bacterial sepsis(n)	37	4		39	2	

As BG values <10 pg/ml were interpreted as negative, we conducted sensitivity and specificity analyses in diagnosing IFD by BG values ≥10 pg/ml, ≥200 pg/ml and ≥400 pg/ml. The diagnostic sensitivity was 68.3%, 49.2% and 33.3%, while the specificity was 75.6%, 91.3% and 96.3%, respectively.

### Platelet Parameters and serum BG in Deceased and Survived Infants with IFD

Among the 63 IFD infants, 13 died due to infections, and 50 infants had a negative blood culture for Candida after the antifungal treatment and were discharged from hospital after recovery. There was no significance in demographical and clinical characteristics between deceased and survived patients (Table 4). However, mortality ratein BG ≥400 pg/ml patients was statistically higher (*p* = 0.02), while no significance was found between the BG ≥200 and BG <200 pg/ml groups (Table 5).

**Table 4:** Birth, clinical characteristics and hematological findings in deceased versus survived IFD at the onset and 14 days after antifungal therapy in survived IFD infants.

Variable		Onset of deceased IFD (n = 13)	Onset of survived IFD (n = 50)	14 days after antifungal therapy in the survived IFD	*P*1 value^a^	*P*2 value^b^
Demographics						
	Male	7(53.8)	27(54.0)	-	0.99	-
	Gestational age (weeks)	30.2(2.3)	30.9(2.3)	-	0.38	-
	Birth weight (g)	1441.3(412.6)	1496.4(445.7)	-	0.69	-
	Age at onset of IFD (days)	20.8(5.9)	21.7(7.1)	-	0.70	-
	Mode of delivery (caesarean)	9(69.2)	31(62.0)	-	0.75	-
Underlying diseases						
	Asphyxia	6(46.2)	15(30.0)	-	0.33	-
	Bacterial infection	1(7.7)	10(20.0)	-	0.43	-
	Necrotizing Enterocolitis	3(23.1)	3(6.0)	-	0.10	-
	Respiratory Distress Syndrome	4(30.8)	7(14.0)	-	0.22	-
	Heart disease	3(23.1)	14(28.0)	-	1.00	-
	Liver injury	2(15.4)	8(16.0)	-	1.00	-
Antecedent neonatal events						
	Invasive Mechanical Ventilation	7(53.8)	14(28.0)	-	0.10	-
	Duration of parenteral nutrition(days)	18.2(6.4)	19.1(6.6)	-	0.73	-
	Fluconazole prophylaxis	5(38.5)	24(48.0)	-	0.54	-
	Presence of CVC	12(92.3)	32(64.0)	-	0.09	-
Hematological findings						
	HB (g/L)	109.2(18.2)	122.9(28.6)	118.5(21.2)	0.11	0.40
	WBC (×10^9^/L)	11.2(6.7)	11.3(6.6)	10.4(4.5)	0.82	0.87
	PC (×10^9^/L)	49.2(43.6)	107.9(85.1)	270.0(137.3)	0.002	<0.001
	PCT (%)	0.06(0.05)	0.15(0.15)	0.32(0.16)	0.005	<0.001
	PDW (fl)	10.6(3.5)	13.9(3.8)	15.8(4.9)	0.07	0.025
	MPV (fl)	13.1(0.9)	12.4(2.3)	12.3(2.0)	0.38	0.016

Data are presented as the number of subjects in each group, with percentages given in parentheses or mean, with SD given in parentheses.

^a^ *P*1 values compares the deceased IFD with the survived IFD.

^b^ *P*2 values compares hematological parameters at the onset day and at the 14^th^ day after antifungal treatment in the survived IFD.

**Table 5:** Hematological parameters and mortality among infants in different BG value groups.

	BG value (pg/ml)		*P* value	BG value (pg/ml)		*P* value
	<200	≥200		<400	≥400	
Survived IFD(n)	27	23	0.32	37	13	0.02
Deceased IFD(n)	5	8		5	8	
HB (g/L)	120.2(27.9)	120.0(27.0)	0.99	121.1(29.4)	119.0(25.2)	0.76
WBC (×10^9^/L)	11.5(5.7)	11.1(7.4)	0.85	12.8(7.3)	9.7(5.3)	0.06
PC (×10^9^/L)	116.1(96.3)	76.1(59.6)	0.10	115.2(93.6)	74.4(60.6)	0.04
PCT (%)	0.14(0.12)	0.13(0.16)	0.60	0.18(0.17)	0.09(0.07)	0.04
PDW (fl)	12.8(3.1)	13.7(4.7)	0.46	13.8(3.9)	12.8(4.0)	0.44
MPV (fl)	12.3(2.8)	12.8(1.0)	0.75	12.2(2.8)	12.9(1.0)	0.48

Data are presented as the number of subjects or mean (SD).

**Fig 2:**
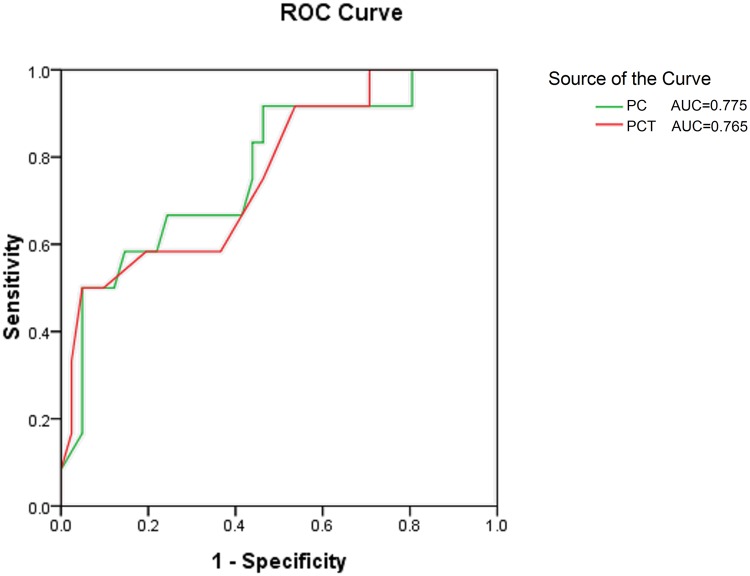
Receiver operating characteristic curves for predicting mortality in IFD infants by platelet count (PC) and plateletcrit (PCT). For each indicator, sensitivity (true positive rate) is plotted against 1-specificity (false positive rate). Accuracy is measured by the area under the ROC curve. The AUCs for PC and PCT were 0.775 (95% CI: 0.629–0.920, p = .002) and 0.765 (95% CI: 0.610–0.921, p = .006), respectively.

We also compared hematological findings on the onset of IFD between the deceased and survived patients. The results showed that only PC, PCT levels were significantly lower in deceased infants (Table 4). We conducted ROC curve analysis to predict mortality in IFD infants. Fig 2 showed the AUCs for PC and PCT were 0.775 (CI: 0.629–0.920, *p* = .002) and 0.765 (CI: 0.610–0.921, *p* = .006), respectively. The best cut-offs for predicting IFD mortality were 39 for PC (sensitivity 62%, specificity 86%) and 0.04 for PCT (sensitivity 50%, specificity 95%), respectively.

### Platelet Parameters and BG after Antifungal Therapy

There were 13 IFD infant deaths due to the infection, and the median duration from the disease onset to death was 10 days (range: 1–20 days). Among the 50 survived IFD infants, the median duration from BG positive to negative was 18 days (range: 7–100 days).

In the 50 survived IFD infants, statistically significant elevations in PC, PCT and PDW levels and reductions in MPV levels were observed after 14 days of antifungal treatment (Table 4).

### The Relationship between Platelet Markers and Serum BG

We compared platelet parameters by BG level cutoff at 200 or 400 pg/ml. Statistically significant differences were observed in both PC (*p* = 0.04) and PCT (*p* = 0.04) levels using the 400 pg/ml cut off. However, there was no significance using the 200 pg/ml cutoff (Table 5).

## Discussion

PC, MPV, PDW and PCT are the often used parameters reflecting platelet function. To our knowledge, our study is the first to show that PC, PCT, PDW and MPV are strong predictors of neonatal IFD. In the present study, we have shown that there are quantitative differences in the platelet response to IFD, bacterial sepsis and preterm control infants. A decrease in PC, PCT or PDW and an increase in MPV may be a marker of IFD, while an increase in PC, PCT or PDW and a decrease in MPV may be a marker for better prognosis of IFD after antifungal therapy. Furthermore, an extremely decrease in PC and PCT could be helpful in differentiating IFD from bacterial sepsis.

In a multicenter analysis of infants weighing less than 1250 g, a PC of less than 150×10^9^/L increased the likelihood of candidemia [20]. Similarly, we used a cutoff value of 119.5×10^9^/L for PC with sensitivity = 0.78 and specificity = 0.95 in diagnosing IFD. Thrombocytes were discovered to be part of the innate immune system with antimicrobial functions [21]. It has been suggested that platelets have immunological functions, and may participate in the interaction between pathogens and host defense [22]. The specific importance of platelets for the pathogenesis of fungal infection may differ between patient groups according to the presence or absence of thrombocytopenia. Besides, PCT, which indicates the total amount of platelets, was correlated positively with platelet count. The decrease in PCT was commonly seen in thrombocytopenia. We also found that PC, PCT levels were significantly decreased in IFD infants, and more extremely so in deceased IFD infants, indicating that changes in PC and PCT levels might be associated with the severity and prognosis of fungal infection.

Bacteria sepsis is another common cause of thrombocytopenia in the NICU. Our study showed that PC values in infants with bacteria sepsis were lower than in preterm control infants, but still higher than in IFD infants. In addition, PCT values in IFD infants were significantly lower than in infants with bacteria sepsis. Consistent with our study findings, Guida et al reported that fungal sepsis caused a significantly greater relative decrease in platelet count from baseline as compared to Gram-positive sepsis [23].

Increased MPV suggests an increased consumption of platelets stimulating the bone marrow to produce and release more platelets in the circulation, because platelets decrease in size as they age, and is suggestive of increased platelet production and/or increased platelet destruction. In 2 different studies with sepsis, high MPV appeared to be prominent features [23, 24]. Platelets with a greater volume are functionally more active and hypersensitive, with a lower threshold for aggregation activity, stronger adhesive capacity and blood stasis power, likely because large platelets contain more dense granules and produce more thromboxane [25]. So, MPV may be another useful indicator for diagnosing IFD.

PDW is the release of large platelets from bone marrow in response to greater demand for platelets. PDW is also a quantitative assessment of platelet size, and volume is of limited usefulness in distinguishing reactive thrombocytosis and essential thrombocythemia. PDW is increased in the presence of platelet anisocytosis [26]. We found that PDW were decreased in IFD infants, contradicting the findings in Patrick`s study on changes in platelet parameters in neonates with bacteremia showing that platelet counts correlated negatively with PDW during the course of infection [27]. We speculated that at the onset of IFD, platelets in peripheral blood were reduced while bone marrow megakaryocytes had not yet been stimulated to release large platelets promptly. Thus, PDW seems to have been decreased in IFD infants. After anti-fungal therapy, bone marrow megakaryocytes were stimulated released large platelets into the peripheral blood leading to increased PDW.

Additionally we found that PC, PCT levels were significantly lower in deceased versus survived IFD infants. A possible reason for marked thrombocytopenia and decreased PCT in deceased IFD infants may be that the severity of thrombocytopenia was directly related to the presence of disseminated intravascular coagulation (DIC). Platelet activation, consumption, and destruction may occur at the endothelial cell surface as a result of thrombin generation and fibrin meshwork formation secondary to coagulation activation [28]. A significant decrease in platelet count in IFD neonates may indicate the presence of DIC which can easily lead to death. So, marked thrombocytopenia may be a warning sign of severe IFD.

In recent studies, elevated serum BG levels were observed in a wide variety of fungi infections including candida [6, 8, 29]. Circulating BG levels as an adjunct diagnostic tool for IFD may be useful in NICU patients among whom IFD and bacterial infection may have similar clinical presentations [8]. The optimal serum BG cut-off value for diagnosing invasive fungal diseases ranged from 10 to140 pg/ml [30, 31]. In our study, we found that the sensitivity in diagnosing IFD was 68.3% by BG ≥10 pg/ml, 49.2% by BG ≥200 pg/ml and 33.3% by BG ≥400 pg/ml while the specificity was 75.6%, 91.3% and 96.3%, respectively. Our results also showed that PC and PCT levels in the BG≥400 pg/ml group were lower compared to the BG<400 pg/ml group. PC and PCT levels might be associated with the prognosis of fungal infection. Low PC and PCT and high BG values were associated with an increased risk of mortality.

It should be emphasized that we did, however, observe significant differences in BG value between IFD and bacterial sepsis. Although bacteraemia has been reported to be related to false positive results in the BG test [32], other studies did not identify bacteraemia as a major cause of false positive results in the BG test [33, 34]. Besides, a BG cutoff >80pg/ml was frequently regarded as positive [33, 35]. In our study, only 2 neonates with bacterial sepsis had a BG value >400mg/dl. Using a high BG cutoff could be more helpful in differentiating IFD neonates from bacterial sepsis.

In conclusion, platelet parameters and BG, which are inexpensive to measure and widely available in routinely collected clinical data, may be useful for the diagnosis and prognosis of neonatal IFD. Moreover, platelet-associated hematological parameters, together with the BG test, can be used as a more efficient supplementary diagnostic tool for IFD.
